# Pathobiological Implications of Mucin (MUC) Expression in the Outcome of Small Bowel Cancer

**DOI:** 10.1371/journal.pone.0086111

**Published:** 2014-04-10

**Authors:** Hiroaki Shibahara, Michiyo Higashi, Chihaya Koriyama, Seiya Yokoyama, Iwao Kitazono, Yasuhiro Kurumiya, Michihiko Narita, Shingo Kuze, Takanori Kyokane, Saburo Mita, Toshiyuki Arai, Takehito Kato, Norihiro Yuasa, Ryuzo Yamaguchi, Hitoshi Kubota, Hideaki Suzuki, Satoshi Baba, Karine Rousseau, Surinder K. Batra, Suguru Yonezawa

**Affiliations:** 1 Department of Palliative Care, Toyota Kosei Hospital, Toyota, Japan; 2 Department of Human Pathology, Field of Oncology, Kagoshima University Graduate School of Medical and Dental Sciences, Kagoshima, Japan; 3 Department of Epidemiology and Preventive Medicine, Kagoshima University Graduate School of Medical and Dental Sciences, Kagoshima, Japan; 4 Department of Surgery, Toyota Kosei Hospital, Toyota, Japan; 5 Department of Pathology, Toyota Kosei Hospital, Toyota, Japan; 6 Department of Surgery, Chutoen General Medical Center, Kakegawa, Japan; 7 Department of Surgery, Chita City Hospital, Chita, Japan; 8 Department of Surgery, Anjo Kosei Hospital, Anjo, Japan; 9 Department of Surgery, Toyohashi Municipal Hospital, Toyohashi, Japan; 10 Department of Surgery, Japanese Red Cross Nagoya Daiichi Hospital, Nagoya, Japan; 11 Department of Surgery, Kasugai Municipal Hospital, Kasugai, Japan; 12 Department of Surgery, Handa City Hospital, Handa, Japan; 13 Department of Diagnostic Pathology, University Hospital Hamamatsu University School of Medicine, Hamamatsu, Japan; 14 Wellcome Trust Centre for Cell-Matrix Research, Faculty of Life Sciences, University of Manchester, United Kingdom; 15 Departments of Biochemistry and Molecular Biology, Eppley Institute for Research in Cancer and Allied Diseases, University of Nebraska Medical Center, Omaha, Nebraska, United States of America; The University of Tokyo, Japan

## Abstract

Mucins have been associated with survival in various cancer patients, but there have been no studies of mucins in small bowel carcinoma (SBC). In this study, we investigated the relationships between mucin expression and clinicopathologic factors in 60 SBC cases, in which expression profiles of MUC1, MUC2, MUC3, MUC4, MUC5AC, MUC6 and MUC16 in cancer and normal tissues were examined by immunohistochemistry. MUC1, MUC5AC and MUC16 expression was increased in SBC lesions compared to the normal epithelium, and expression of these mucins was related to clinicopathologic factors, as follows: MUC1 [tumor location (p = 0.019), depth (p = 0.017) and curability (p = 0.007)], MUC5AC [tumor location (p = 0.063) and lymph node metastasis (p = 0.059)], and MUC16 [venous invasion (p = 0.016) and curability (p = 0.016)]. Analysis of 58 cases with survival data revealed five factors associated with a poor prognosis: poorly-differentiated or neuroendocrine histological type (p<0.001), lymph node metastasis (p<0.001), lymphatic invasion (p = 0.026), venous invasion (p<0.001) and curative resection (p<0.001), in addition to expression of MUC1 (p = 0.042), MUC5AC (p = 0.007) and MUC16 (p<0.001). In subsequent multivariate analysis with curability as the covariate, lymph node metastasis, venous invasion, and MUC5AC and/or MUC16 expression were significantly related to the prognosis. Multivariate analysis in curative cases (n = 45) showed that SBC with MUC5AC and/or MUC16 expression had a significantly independent high hazard risk after adjusting for the effects of venous invasion (hazard ratio: 5.6, 95% confidence interval: 1.8–17). In conclusion, the study shows that a MUC5AC-positive and/or MUC16-positive status is useful as a predictor of a poor outcome in patients with SBC.

## Introduction

Small bowel carcinoma (SBC) is a rare malignancy, in contrast to colorectal carcinoma. A surgical approach is mainly used to treat SBC [1]–[6], but many patients have a poor outcome after curative resection. Lymph node metastasis [1]–[3], distant metastasis [6], primary tumor status [2], [6] and tumor differentiation [1] have been reported as prognostic factors in SBC.

Mucins are high molecular weight glycoproteins in which the core proteins are modified by O-glycoside-linked oligosaccharides [7]. Eighteen core human mucins (MUC1–MUC8, MUC12, MUC13, MUC15–17 and MUC19–21) have been identified. The first cloned mucin, MUC1, is an important human tumor antigen, ranking second after WT1 [8]. Using immunohistochemistry (IHC), we have shown that MUC1 and/or MUC4 expression is related to a poorer prognosis, whereas MUC2 expression is associated with a better prognosis in various human tumors [7], [9]. Aberrant expression of MUC3, MUC4, MUC5AC and MUC6 has been described in pancreatic intraepithelial neoplasia [10], [11], and we recently reported that MUC16 is a candidate as a poor prognostic factor in cholangiocarcinoma [12].

To date, only two articles have discussed mucins in SBC [13], [14] and the clinical significance of mucin expression in SBC is unknown. Therefore, the aim of the present study was to investigate whether expression of mucins (MUC1, MUC2, MUC3, MUC4, MUC5AC, MUC6 and MUC16) has prognostic significance in patients with SBC using specimens obtained from surgical departments at multiple hospitals.

## Materials and Methods

### Patients and tissue specimens

Between 1973 and 2011, 60 resected specimens of SBC were collected from Toyota Kosei Hospital, Chutoen General Medical Center, Chita City Hospital, Anjo Kosei Hospital, Toyohashi Municipal Hospital, Japanese Red Cross Nagoya Daiichi Hospital, Kasugai Municipal Hospital, Handa City Hospital, and Kagoshima-shi Medical Association Hospital. Cancers of the ampulla of Vater or possible metastatic cancer were excluded from the study. The patients were 28 men and 32 women with an age range of 34 to 90 (mean 65) years old. The tumor locations were the duodenum (24 cases), jejunum (20), ileum (14), and not specified (2). This study was conducted in accordance with the guiding principles of the Declaration of Helsinki. Informed, written consent was obtained from 6 patients, and was approved by the Ethics Committee of Kagoshima-shi Medical Association Hospital (KMAH 2011-02-02). For the other patients without informed consent, the Institutional Review Board of Toyota Kosei Hospital (22-ST04), the Ethics Committees of Toyohashi Municipal Hospital (43-2011), Japanese Red Cross Nagoya Daiichi Hospital (26-2013), Kasugai Municipal Hospital (157-2013), Chutoen General Medical Center, Chita City Hospital and Handa City Hospital, and the hospital director of Anjo Kosei Hospital gave us their approvals to use the resected specimens (No specified number in the latter four hospitals), under the strict condition of privacy protection in relation to personal information of the patients.

The surgical procedures were partial resection of the small intestine (31 cases), pancreaticoduodenectomy (8), subtotal stomach-preserving pancreaticoduodenectomy (7), pylorus-preserving pancreaticoduodenectomy (4), ileocecal resection (6), right hemicolectomy (3), and tumor resection (1). Pancreaticoduodenectomies in 19 cases were performed to guarantee a secure surgical margin and sufficient lymph node dissection because the tumors in the duodenum were located near the ampulla of Vater. We confirmed that all resected specimens were small intestinal carcinomas using macroscopic and microscopic pathological findings. Lymph node dissection was performed in 55 cases, not performed in 3 cases, and this information was unknown in 2 cases. Forty-six cases underwent curative resection, 11 cases received non-curative resection because of distant metastasis found in the operation, and the details were unknown for 3 cases. Among the 60 patients, 23 died of primary disease and one died of metachronous primary advanced gastric cancer with carcinomatous peritonitis. Overall survival was analyzed in 58 patients, but was unknown in two patients.

### Immunohistochemistry

All specimens were fixed in formalin, embedded in paraffin and cut into 4-µm thick sections for IHC, in addition to hematoxylin and eosin (HE) staining. MUC1 was detected by monoclonal antibody (MAb) DF3 (mouse IgG, Toray-Fuji Bionics, Tokyo, Japan), MUC2 by MAb Ccp58 (Novocastra), MUC3 by MAb mMUC3-1 (generated by K. Rousseau and D. M. Swallow), MUC4 by MAb 8G7 (generated by S. K. Batra), MUC5AC by MAb CLH2 (Novocastra), MUC6 by MAb CLH5 (Novocastra), and MUC16 by MAb OC125 (Acris Antibodies GmbH). IHC was performed by the immunoperoxidase method, as follows. Antigen retrieval was performed using CC1 antigen retrieval buffer (pH8.5, EDTA 100°C 30 min, Ventana Medical Systems, Tucson, AZ, USA). The sections were incubated with a primary antibody (DF3 diluted 1∶50, 37°C, 32 min; Ccp58 diluted 1∶200, 37°C, 24 min; 8G7 diluted 1∶3000, 37°C, 32 min; CLH2 diluted 1∶100, 37°C, 24 min; CLH5 diluted 1∶100, 37°C, 24 min; OC125 diluted 1: 100, 37°C, 24 min) in phosphate-buffered saline pH 7.4 (PBS) with 1% bovine serum albumin and stained on a Benchmark XT automated slide stainer using a diaminobenzidine detection kit (ultraView DAB, Ventana Medical Systems).

For MUC3 staining, the sections were treated at 100°C for 10 min in 0.01 M citrate buffer at pH 6.0 and then reduced with 0.01 M dithiothreitol in 0.1 M Tris/HCl buffer (pH 8.0) for 30 min at room temperature and alkylated with 0.025 M iodoacetamide in 0.1 M Tris/HCl buffer (pH 8.0) for 30 min [10], [15]. They were then incubated with mMUC3-1 at 4°C for 16 h and stained by the avidin-biotin complex method. Reaction products were not present when hybridoma culture medium, normal mouse serum, or PBS was used instead of the primary antibodies.

### Scoring of the staining results

Three blinded investigators (S.H. M.H. and S.Y.) evaluated the IHC staining data independently. When the evaluation differed among the three, a final decision was made by consensus. The results were evaluated based on the percentage of positively stained carcinoma cells. We evaluated staining of the cytoplasm and cell membrane, and the carcinoma cells were considered to be positive when at least one of these components was positive. A tumor was considered positive if more than 5% of carcinoma cells were stained, according to our previous studies using 5% as the cutoff for mucin expression [16]–[21].

### Statistical analysis

The prevalence of expression of each mucin was compared between cancer lesions and normal epithelium using a chi-square test. Associations between mucin expression profiles and clinicopathologic factors were also examined by chi-square test. For survival analysis, a log-rank test was used to select mucins that were significantly related to prognosis. A Cox proportional hazard analysis was used to estimate hazard ratios (HRs) and corresponding 95% confidence intervals (CIs) in adjusting for the effects of other clinicopathologic factors. All reported p values are two-sided and p<0.05 was considered to be significant.

## Results

### Expression profiles of mucin antigens

**Figure 1:**
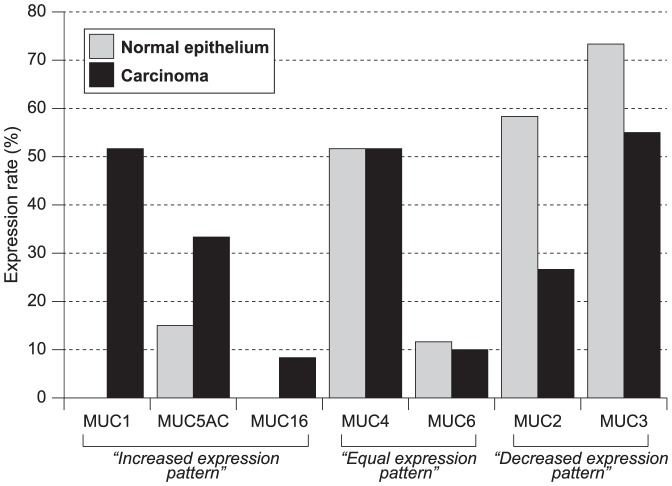
The expression rate of mucins in tissue. MUC1, MUC5AC and MUC16 showed increased expression; MUC4 and MUC6 showed equal expression; and MUC2 and MUC3 showed decreased expression in SBC compared to normal epithelium.

In the normal epithelium of the small intestine, none of the 60 cases showed expression of MUC1 (0%, 0/60) or MUC16 (0%, 0/60), but some expressed MUC2 (58.3%, 35/60), MUC3 (73.3%, 44/60), MUC4 (51.7%, 31/60), MUC5AC (15%, 9/60) and MUC6 (11.7%, 7/60) (Figure 1, Table S1). MUC6 was also expressed in duodenal Brunner's glands in most cases. MUC2, MUC3 and MUC4 showed higher expression in ileum (p = 0.035), jejunum (p = 0.011), and ileum (p = 0.002), respectively, compared to other sites (Figure 1, Table S1).

The expression rates in cancer lesions (more than 5% of carcinoma cells stained) were MUC1, 51.7% (31/60); MUC2, 26.7% (16/60); MUC3, 55% (33/60); MUC4, 51.7% (31/60); MUC5AC, 33.3% (20/60); MUC6, 10% (6/60) and MUC16, 8.3% (5/60) (Figure 1).

### Changes of MUC expression from normal epithelium to carcinomas

Compared to the normal epithelium, MUC1 (p<0.001), MUC5AC (p = 0.019) and MUC16 (p = 0.022) expression was significantly increased in SBC; MUC4 and MUC6 expression showed equal expression; and MUC2 (p<0.001) and MUC3 (p = 0.036) expression was significantly decreased in SBC.

**Figure 2:**
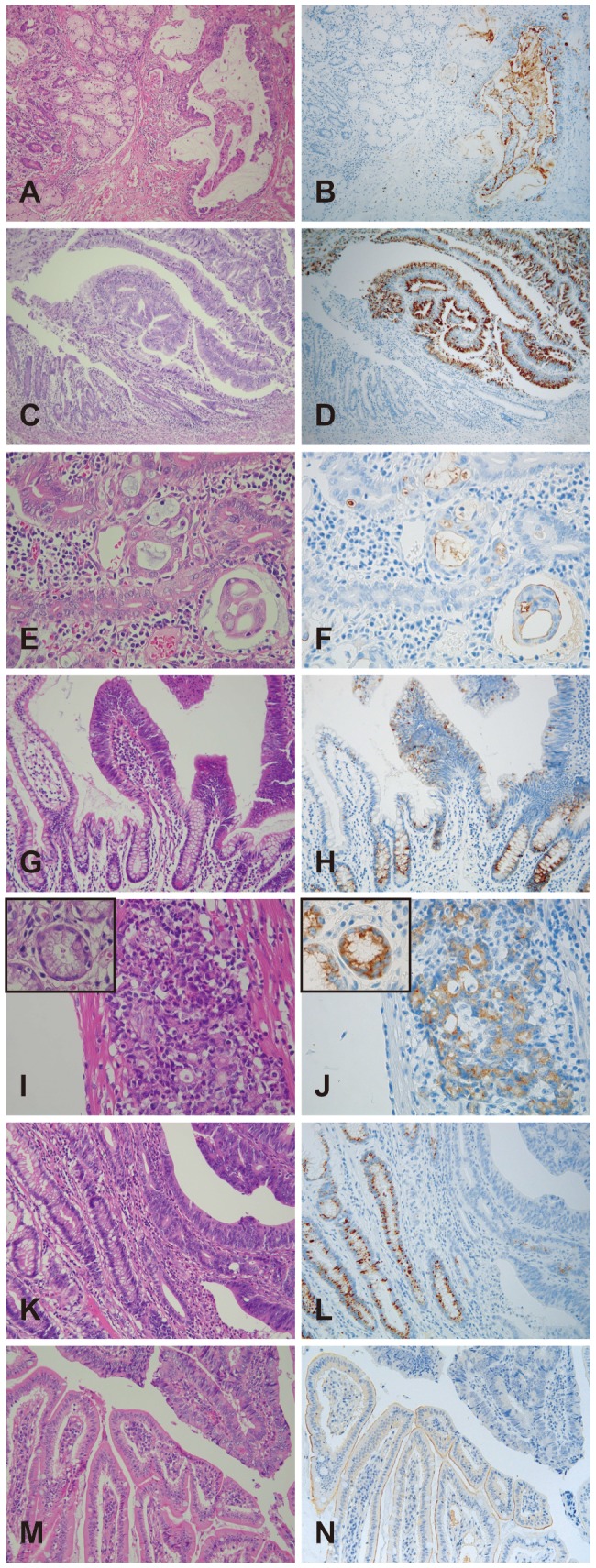
Analysis of the expression of mucins by immunohistochemistry. In mucins with increased expression in SBC, MUC1 showed apical and cytoplasmic expression in cancer cells, but not in the normal epithelium (A and B); MUC5AC showed cytoplasmic expression in cancer cells, but not in the normal epithelium (C and D); and MUC16 showed apical expression in cancer cells, but not in the normal epithelium (E and F). In mucins with equal expression in SBC, MUC4 showed cytoplasmic expression in normal epithelium and cancer cells (G and H); and MUC6 showed cytoplasmic expression in normal epithelium (insets) and cancer cells (I and J). In mucins with decreased expression in SBCs, MUC2 showed cytoplasmic expression in normal epithelium, but not in cancer cells (K and L); and MUC3 showed apical expression in normal epithelium, but not in cancer cells (M and N).

Representative mucin expression patterns in the normal epithelium and cancer tissues are shown in Figure 2. Among mucins with increased expression in SBC, MUC1 (Figures 2A and 2B) showed apical and cytoplasmic expression in carcinoma cells, but not in normal epithelium; MUC5AC (Figures 2C and 2D) showed cytoplasmic expression in carcinoma cells, but not in normal epithelium; and MUC16 (Figures 2E and 2F) showed apical expression in carcinoma cells, but not in normal epithelium. For mucins with equal expression in SBC and normal tissue, MUC4 (Figures 2G and 2H) and MUC6 (Figures 2I and 2J) both showed cytoplasmic expression in carcinoma cells and normal epithelium (insets). Among mucins with decreased expression in SBC, MUC2 (Figures 2K and 2L) showed supranuclear expression in normal epithelium, but not in carcinoma cells; and MUC3 (Figures 2M and 2N) showed apical expression in normal epithelium, but not in carcinoma cells.

### Relationship between MUC expression in cancer cells and clinicopathologic features

Relationships between mucin expression and clinicopathologic features are summarized in Table S2. MUC1 expression was related to tumor location (high for oral side, p = 0.019), invasion depth (higher for deeper than pSS (pT3), p = 0.017), venous invasion (high for positive venous invasion, p = 0.038), and curability (high for non-curative resection, p = 0.007). MUC2 expression was related to tumor location (high for anal side, p = 0.034), negative lymphatic invasion (p = 0.041) and histological type (high for mucinous carcinoma, p = 0.005). MUC4 expression was related to tumor location (high for anal side, p = 0.012). MUC5AC expression was marginally related to tumor location (p = 0.063) and lymph node metastasis (p = 0.059). MUC6 expression was related to lymph node metastasis (high for positive lymph node metastasis, p = 0.021). MUC16 expression was related to venous invasion (high for positive venous invasion, p = 0.016) and curability (high for non-curative resection, p = 0.016).

### Relationship of clinicopathological factors or mucin expression with survival period

**Figure 3:**
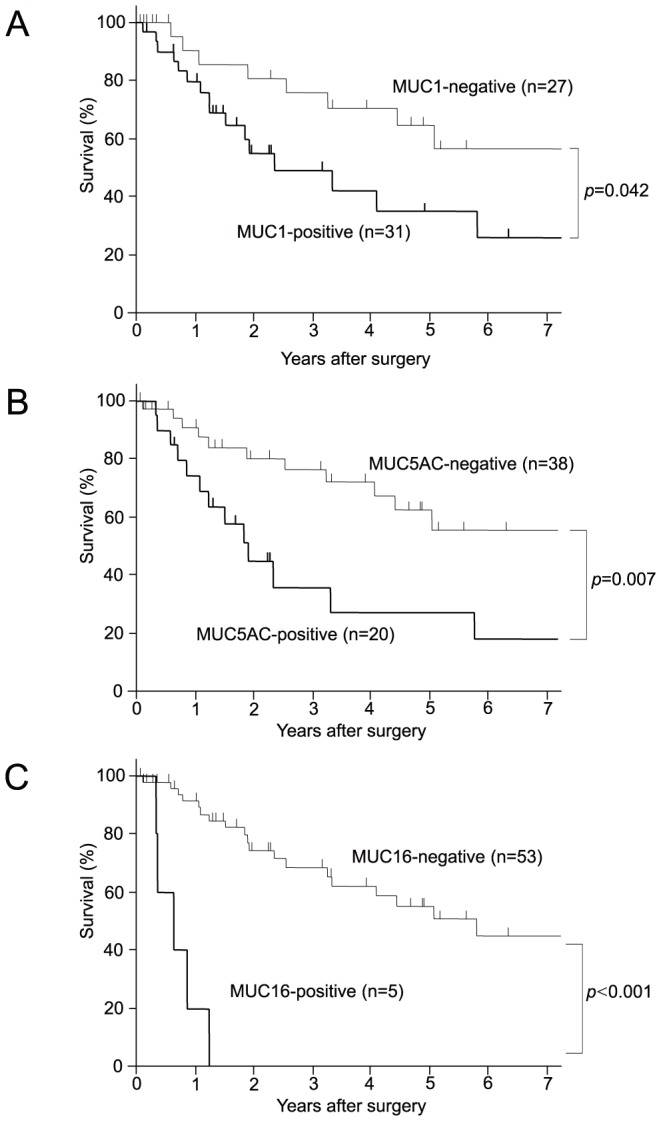
The cumulative survival rates of patients with SBC. The expression of MUC1 (A), MUC5AC (B) and MUC16 (C) were poorer than those of patients without expression of MUC1 (p = 0.042), MUC5AC (p = 0.007) and MUC16 (p<0.001), respectively. Survival data were calculated using the Kaplan-Meier method.

Information on survival was retrieved for 58 cases. The overall 5-year survival rate and the median survival period were 49.7% and 1.9 years (95% CI: 1.3–3.3), respectively (data not shown). A log-rank test showed that histological type (poorly differentiated or neuroendocrine) (p<0.001), positive lymph node metastasis (p<0.001), positive lymphatic invasion (p = 0.026), positive venous invasion (p<0.001), and non-curative resection (p<0.001) were significantly related to a poorer prognosis (Table S3). Expression of MUC1 (p = 0.042), MUC5AC (p = 0.007) and MUC16 (p<0.001) was also significantly related to a poorer prognosis (Figure 3, Table S3). There was no correlation between expression of other mucins (MUC2, MUC3, MUC4, and MUC6) and survival.

### Multivariate analysis of prognostic factors

The above analysis identified MUC1, MUC5AC and MUC16 as candidate prognostic factors in SBC, in addition to five clinicopathologic factors: histological type, lymph node metastasis, lymphatic invasion, venous invasion and curative resection. Since the sample size (n = 58) was too small to estimate the hazard risk using these clinicopathologic factors as covariates in the same model and colinearity among these five factors is likely, curability was chosen as a covariate (Table 1). SBC cases with MUC5AC or MUC16 expression showed significantly worse prognoses. MUC16 had the highest HR (HR:10, 95% CI: 2.8–39), but there were only five MUC16-positive cases.

**Table 1:** Survival analysis using Cox proportional hazard models* with incorporation of curability.

		D/T	P-y	HR	95% CI
**Histological type**	well, mod	19/49	166.7	1.0	reference
	poor, NEC	5/6	4.1	8.0	2.5–26
	muc	0/3	9.1	-	-
**Lymph node metastasis**	Negative	7/26	110.8	1.0	reference
	Positive	16/28	50.7	4.0	1.6–11
**Lymphatic invasion**	Negative	3/18	53.8	1.0	reference
	Positive	21/40	126.1	2.4	0.7–8.7
**Venous invasion**	Negative	8/29	127.5	1.0	reference
	Positive	16/29	52.4	3.7	1.4–10
**MUC1**	Negative	8/27	100.6	1.0	reference
	Positive	16/31	79.4	1.7	0.6–4.6
**MUC5AC**	Negative	11/38	133.1	1.0	reference
	Positive	13/20	46.8	2.5	1.1–6.0
**MUC16**	Negative	19/53	176.5	1.0	reference
	Positive	5/5	3.4	10	2.8–39
**MUC1/MUC5AC**	MUC1(−) and MUC5AC(−)	7/24	83.3	1.0	reference
	MUC1(+) and/or MUC5AC(+)	17/34	96.6	1.6	0.6–4.3
**MUC1/MUC16**	MUC1(−) and MUC16(−)	8/27	100.6	1.0	reference
	MUC1(+) and/or MUC16(+)	16/31	79.4	1.7	0.6–4.6
**MUC5AC/MUC16**	MUC5AC(−) and MUC16(−)	9/36	131.3	1.0	reference
	MUC5AC(+) and/or MUC16(+)	15/22	48.6	3.6	1.5–8.8

*Curability was included in all the statistical models.

NA: not available, D/T: Deaths/Total, P-y: Person-year, HR: Hazard ratio, 95% CI: 95% confidence interval.

Since MUC1, MUC5AC and MUC16 showed increased expression in SBC lesions and were related to a poorer prognosis, the effect of these mucins in combination was also examined. SBC cases with MUC5AC and/or MUC16 expression had a significantly worse prognosis (HR: 3.6, 95% CI: 1.5–8.8) (Table 1). This association remained after adjustment for the effect of venous invasion (HR: 4.5, 95% CI: 1.8–11), but abated after adjustment for lymph node metastasis (HR: 2.4, 95% CI: 0.9–6.2) (data not shown).

For clinical applications, further analyses were conducted in a subgroup of 45 patients treated by curative resection. One case without outcome data was excluded from the total of 46 patients treated by curative resection (Table S2). In the 45 cases, MUC5AC and/or MUC16 expression was a significant independent high hazard risk after adjustment for the effect of venous invasion as a covariate (Model 1 in Table 2, HR: 5.6, 95% CI: 1.8–17), but not for the effect of lymph node metastasis as a covariate (Model 2 in Table 2, HR: 2.7, 95% CI: 0.9–8.8) (Table 2). The expression profiles of MUC2 and MUC3 were also considered, since these mucins had decreased expression in cancer lesions. There was no significant change in the hazard risk with MUC2 or MUC3 expression, although there were no deaths in MUC5AC-negative, MUC16-negative and MUC3-positive cases (Table 2).

**Table 2:** Survival analysis for curative cases (n = 45) using a Cox proportional hazard model*.

				Model 1*1		Model 2*2	
		D/T	P-y	HR	95% CI	HR	95% CI
**MUC5AC**	Negative	7/31	123.2	1.0	ref.	1.0	ref.
	Positive	8/14	40.3	3.4	1.2–9.8	1.6	0.5–4.7
**MUC16**	Negative	13/43	161.6	1.0	ref.	1.0	ref.
	Positive	2/2	1.9	7.3	1.3–42	6.4	1.1–36
**MUC5AC/MUC16**	MUC5AC(−) and MUC16(−)	5/29	121.3	1.0	ref.	1.0	ref.
	MUC5AC(+) and/or MUC16(+)	10/16	42.2	5.6	1.8–17	2.7	0.9–8.8
**MUC5AC/MUC16/MUC2**	MUC5AC(−) and MUC16(−), and MUC2(+)	2/11	32.5	1.0	ref.	1.0	ref.
	MUC5AC(−) and MUC16(−), and MUC2(−)	3/18	88.8	0.6	0.1–3.9	0.3	0.04–2.3
	MUC5AC(+) and/or MUC16(+), and MUC2(+)	0/1	8.5	NA		NA	
	MUC5AC(+) and/or MUC16(+), and MUC2(−)	10/15	33.7	4.8	1.0–23	1.7	0.3–10
**MUC5AC/MUC16/MUC3**	MUC5AC(−) and MUC16(−), and MUC3(+)	0/13	80.7	NA		NA	
	MUC5AC(−) and MUC16(−), and MUC3(−)	5/16	40.6	1.0	ref.	1.0	ref.
	MUC5AC(+) and/or MUC16(+), and MUC3(+)	6/10	24.2	3.1	0.8–12	2.0	0.6–7.1
	MUC5AC(+) and/or MUC16(+), and MUC3(−)	4/6	18.0	1.8	0.5–7.0	0.6	0.1–2.7

*1 The status of venous invasion was included as a covariate in models 1.

*2 The status of lymph node metastasis was included as a covariate in models 2.

NA: not available, D/T: Deaths/Total, P-y: Person-year, HR: Hazard ratio, 95% CI: 95% confidence interval, ref.: reference.

## Discussion

There have been two previous analyses of mucin expression in SBC tissues in 30 cases by Zang et al. [13] and in 23 cases by Lee et al. [14] In the present study of 60 cases, we found that increased expression of MUC1, MUC5AC and MUC16 in SBCs was related to poor prognostic factors such as deeper invasion, venous invasion, lymph node metastasis, and cases in which only non-curative resection was possible. For mucins with equal expression in SBC lesions and normal tissue, MUC4 expression was not related to any prognostic factors, but MUC6 was related to lymph node metastasis. Among mucins with decreased expression in SBC, MUC2 expression was related to negative lymphatic invasion (a favorable prognostic factor), while MUC3 was not related to any clinicopathologic factors.

Analysis of mucin expression in 58 patients with information on survival showed that increased expression of MUC1, MUC5AC and MUC16 in SBC was significantly related to a poorer prognosis. Therefore, MUC1, MUC5AC and MUC16 were identified as candidate prognostic factors and subjected to multivariate survival analysis. MUC1 is overexpressed and aberrantly glycosylated in most cancers, and elevation of the MUC1 level plays an important role in tumor invasion and metastasis [7], [9]. A log-rank test in the 58 patients showed that MUC1 expression was related to poor survival. However, in multivariate analysis, MUC1 was not related to prognosis. Thus, compared with many other human neoplasms [7], [9], MUC1 expression seems to be of little significance in SBC. In contrast to MUC1, multivariate analysis showed that MUC5AC or MUC16 expression was significantly related to a worse prognosis in SBC. This relationship has not been examined previously. MUC5AC or MUC16 expression was significantly related to poor survival in patients with SBC, and in 45 patients who underwent curative resection, cases with MUC5AC and/or MUC16 expression had a significantly higher hazard risk factor.

Overexpression of MUC5AC has been associated with poor prognosis of lung cancer, cholangiocarcinoma and pancreatic cancer [22]–[24]. However, till date the molecular mechanism of its functioning is still obscure. The knockdown studies revealed that MUC5AC overexpression in tumor cells is associated with increased growth, adhesion, invasion of tumor cells and increased metastatic tendency [25]–[27]. Further, it is associated with lower infiltration of B cells and neutrophils at metastatic sites [26]. And, Inaguma et al., observed that GLI1-upregulated MUC5AC facilitates the migration and invasion of pancreatic cancer cells through the attenuation of E-cadherin-mediated intercellular adhesion [28]. Thus by suppressing immune infiltration and enhancing adhesion and invasion of tumor cell, MUC5AC might be implicated in poor prognosis of the patients with SBC.

MUC16 is also overexpressed in many malignancies, including ovarian, pancreatic and breast cancers [29]–[31] and its overexpression is associated with poor prognosis. MUC16 is also known as a potential biomarker for the following ovarian cancer after various therapies. Lakshmanan et al. have recently established the functional role of MUC16 in the proliferation of breast cancer cells [31]. Haridas et al. have also demonstrated that the increased expression of MUC16 in progression of pancreatic cancer [30]. Similarly, in the present study we have observed overexpression of MUC16 in 5 of 60 SBC tissues (8.3%) compared to normal small intestine (0%). The number of MUC16 positive cases is small, but the MUC16 expression was significantly related to poor prognosis of the patients with SBC.

MUC4 and MUC6 showed equal expression in SBC and normal tissue. MUC4 expression is a poor prognostic factor in various human neoplasms [7], [9], but we found no correlation between expression of MUC4 and survival in patients with SBC. Thus, compared with neoplasms of other organs, MUC4 expression is of little significance in SBCs, similarly to MUC1. MUC6 is a useful marker for classification of pancreatobiliary neoplasms [32], [33]. In SBCs, however, MUC6 expression had no impact on survival, although it was related to lymph node metastasis.

Expression profiles of MUC2 and MUC3 were also considered, since these mucins showed decreased expression in SBC. Our previous studies showed that MUC2 expression is related to a good prognosis in neoplasms of the pancreas, bile duct and stomach [7], [9]. MUC3 expression is associated with a poor prognosis in gastric cancer [34]; however, little is currently known about the functional role of MUC3 in cancer pathology [7]. In the present study, there was no significant change in hazard risks with MUC2 or MUC3 expression. It is important to note that there were no deaths in SBC patients with a MUC5AC-negative, MUC16-negative and MUC3-positive expression profile.

In conclusion, the results of this study demonstrate that a MUC5AC-positive and/or MUC16-positive mucin expression pattern is a useful marker to predict a poor outcome in patients with SBC. This pattern differs from the expression patterns involving MUC1, MUC2 or MUC4 that are related to a poor prognosis in neoplasms of other organs.

## Supporting Information

Table S1 MUC expression in the normal epithelium.
(XLS)

Table S2 Relationship of expression of MUC1, MUC2, MUC3, MUC4, MUC5AC, MUC6 and MUC16 and clinicopathological features of small bowel carcinoma (n = 60).
(XLS)

Table S3 Survival in patients with small bowel carcinoma by Log-Rank test (n = 58).
(XLS)
